# Emotional Eating in Adolescence: Effects of Emotion Regulation, Weight Status and Negative Body Image

**DOI:** 10.3390/nu13010079

**Published:** 2020-12-29

**Authors:** Lenka H. Shriver, Jessica M. Dollar, Susan D. Calkins, Susan P. Keane, Lilly Shanahan, Laurie Wideman

**Affiliations:** 1 Department of Nutrition, University of North Carolina at Greensboro (UNCG), Greensboro, NC 27402, USA; 2 Department of Human Development and Family Studies, UNCG, Greensboro, NC 27402, USA; jmdollar@uncg.edu; 3 Office of Research and Engagement, UNCG, Greensboro, NC 27402, USA; sdcalkin@uncg.edu; 4 Department of Psychology, UNCG, Greensboro, NC 27402, USA; spkeane@uncg.edu; 5 Jacobs Center for Productive Youth Development, University of Zurich, 8050 Zurich, Switzerland; lilly.shanahan@uzh.ch; 6 Department of Kinesiology, UNCG, Greensboro, NC 27402, USA; l_widema@uncg.edu

**Keywords:** child emotion regulation, emotional eating, adolescence, weight, body image

## Abstract

Emotional eating is associated with an increased risk of binge eating, eating in the absence of hunger and obesity risk. While previous studies with children and adolescents suggest that emotion regulation may be a key predictor of this dysregulated eating behavior, little is known about what other factors may be influencing the link between emotional regulation and emotional eating in adolescence. This multi-method longitudinal study (*n* = 138) utilized linear regression models to examine associations between childhood emotion regulation, adolescent weight status and negative body image, and emotional eating at age 17. Emotion regulation predicted adolescent emotional eating and this link was moderated by weight status (*β* = 1.19, *p* < 0.01) and negative body image (*β* = −0.34, *p* < 0.01). Higher engagement in emotional eating was predicted by lower emotional regulation scores among normal-weight teens (*β* = −0.46, *p* < 0.001) but not among overweight/obese teens (*β* = 0.32, *p* > 0.10). Higher scores on emotion regulation were significantly associated with lower emotional eating at high (*β* = −1.59, *p* < 0.001) and low (*β* = −1.00, *p* < 0.01) levels of negative body image. Engagement in emotional eating was predicted by higher negative body image among overweight/obese teens only (*β* = 0.70, *p* < 0.001). Our findings show that while better childhood emotion regulation skills are associated with lower emotional eating, weight status and negative body image influence this link and should be considered as important foci in future interventions that aim to reduce emotional eating in adolescence.

## 1. Introduction

Emotional eating is defined as “the use of food as a coping mechanism when faced with stress or negative feelings” in the current literature [1,2,3]. While most of the previous studies report on emotional eating in response to negative affect, such as sadness or anger, a recent systematic review suggests that individuals may also engage in emotional eating when they are experiencing positive feelings, such as joy or excitement [1,4]. Regardless of the type of affect individuals respond to, emotional eating has been associated with greater adiposity and higher Body Mass Index (BMI) [5,6]. Emotional eating has also been linked to behaviors that may contribute to increased risk of obesity overtime, including binge eating and loss of control over eating [7,8]. Emotional eating was also identified as a key mediator between depression and obesity [9]. Furthermore, emotional eating is strongly correlated with poor dietary intake outcomes, including higher intakes of added sugars and fat. These findings are not surprising since emotional eaters tend to turn to high-energy and low-nutrient density foods in their response to their emotional feelings [10,11,12].

Among young children, decreased appetite is a typical response to stress [13]. However, research suggests that more than 60% of 5–13-year-old children report eating in response to mood states [14]. More importantly, emotional eating is believed to increase substantially between childhood and adolescence [9,13,15,16]. Some argue that this increase is due to pubertal hormones that begin to influence appetite and body weight [15]. Given the strong associations between emotional eating, poor dietary outcomes, and higher obesity risk [5,6,17], further research is warranted to identify and better understand predictors and correlates of emotional eating in adolescence.

### 1.1. Emotional Eating and Emotion Regulation

Emotion regulation is a form of self-regulation that has been implicated not only in obesity research, but also in studies related to depression and eating behaviors [18,19,20,21,22,23]. Emotion regulation is defined as behaviors, skills, and/or strategies that are used to modulate emotional experiences and expressions, with these behaviors and/or skills being either conscious or unconscious, and either automatic or effortful [24]. Poor emotion regulation in children has been identified as a predictor of high-risk behaviors (i.e., substance abuse) [25], mental health problems [26], and a higher risk of becoming overweight or obese [20,22]. In a conceptual framework developed by Aparicio et al. [17], the link between emotional dysregulation and obesity is mediated by engagement in emotional eating.

A number of previous studies have found associations between poor emotion regulation and dysregulated eating behaviors. While many of these findings come from samples of adults [4,27], a few studies have examined the associations between emotion regulation and emotional eating among children and adolescents [5,28,29,30]. For instance, Harrist et al. [28] found that poor emotion regulation skills, specifically reactivity to anger and worry, predicted greater increases in emotional eating between second and third grade in a sample of 782 children. Furthermore, our own recent work utilizing a path analyses in a community sample of adolescents found that emotion regulation predicted adolescent adiposity via the mediating effect of emotional eating [5]. While there is growing evidence that emotion regulation influences eating behaviors among children and adolescents [5,28], it is likely there are important personal, physical and/or social factors that also influence the degree to which some adolescents may engage in more emotional eating than others.

### 1.2. Weight Status, Body Image and Emotional Eating

Adolescence represents a unique developmental period that is marked by increased independence in daily decisions, and the growing influence of peers over parents on one’s attitudes and beliefs, including those related to overall self-esteem and body image [31,32,33,34]. Thompson et al. [35] defines body image as one’s subjective assessment of his/her own appearance. Studies indicate that body dissatisfaction begins to increase as children get older and poor body esteem increasingly becomes a risk factor for a variety of unhealthy behaviors, including dieting, disordered eating and substance abuse during adolescence [31]. As such, negative body image has emerged as an important contributor to both physical and psychosocial functioning among adolescents and young adults in previous research [36,37,38,39]. In fact, higher BMI predicts body dissatisfaction [40] and overweight and obese individuals tend to have higher levels of body dissatisfaction compared to their normal-weight peers [41]. Furthermore, other studies have shown that body dissatisfaction predicts overweight/obesity [42]. Since body dissatisfaction encompasses negative feelings related to one’s body and/or appearance [43,44], it may trigger eating in the absence of hunger as a response to the negative feelings. This notion is supported by findings from previous studies that found higher levels of emotional eating among overweight and obese children compared to their normal weight peers [30,43]. Therefore, it is likely that emotion regulation, weight status and negative body image interact to affect engagement in emotional eating. To our knowledge, previous research has not directly examined these associations in children or adolescents.

### 1.3. Rationale and Study Aims

Adolescence is a unique developmental period in which individuals continue to refine lifestyle habits that influence their long-term health outcomes [1,5,28,45]. Because emotional eating is a dysregulated behavior linked with greater obesity risk and a number of additional negative health-related outcomes, it is critical to better understand the factors that may predispose some adolescents to emotional eating while keeping others protected. Since individuals’ ability to regulate their emotions is largely developed and relatively stable by middle-childhood [46,47], research is warranted to examine whether emotion regulation deficits in middle-childhood may represent an early intervention target for decreasing the risk of future emotional eating during adolescence. Given the increasing emotional and psychological stress related to weight status and body image that children experience [44], adolescence is the ideal developmental period to study these factors. The purpose of the current study was to examine associations between childhood emotion regulation, adolescent weight status and negative body image, and adolescent emotional eating in a community sample of adolescence. The current study utilized a multi-method longitudinal study to: (1) examine whether childhood emotion regulation at age 7 is associated with emotional eating at age 17; (2) examine whether weight status in mid-adolescence (age 15) moderates the association between age 7 emotion regulation and emotional eating at age 17; (3) examine whether age 15 negative body image moderates the association between emotion regulation and emotional eating; and (4) whether age 17 weight status moderates the association between age 15 negative body and age 17 emotional eating.

## 2. Materials and Methods

Data for the current analyses came from an original sample of 445 who began to participate in a longitudinal study called the RIGHT Track study, and later as the RIGHT Track Health project [48,49]. Participants were recruited when they were 2 years of age in childcare centers, the County Health Department, and the local Women, Infants, and Children program. Participants and their mothers participated in the ongoing longitudinal study beginning at age 2 through adolescence. The original sample (at age 2) was racially/ethnically diverse; 64.1% of the children were European American, 29.9% African American, 3.6% biracial, and 2.4% identified as other race/ethnicity. Additional details about sample recruitment and the child and adolescent health assessments are described elsewhere [48,49].

The study was approved by the University Institutional Review Board at the University of North Carolina Greensboro, NC, USA. Data for the current study came from data collection at the following 3 time points: age 7, age 15, and age 17. Each visit included participants (or their parents when appropriate) completing questionnaires, and a variety of anthropometric, physiological and metabolic assessments. Questionnaire packets were mailed to the parents and/or participants who could not complete a lab visit each of the time points. Parents provided consent and the children provided assent, when appropriate, prior to any data collection. The demographic and socioeconomic characteristics [i.e., socioeconomic status (SES)] of the sample were obtained from data collected at the 15-year visit. Participants’ SES was calculated using data from the 15-year visit by calculating the Hollingshead index [49,50]. The main variables and measures relevant for the current study are described below.

Emotional eating was measured using the Three Factor Eating Questionnaire (TFEQ) and completed by the adolescent participants as part of their 17-year visit [51]. The TFEQ questionnaire was validated for ages 12 and up and has been used extensively in previous research with adolescents while demonstrating good reliability [51,52]. The emotional eating subscale (3 items; α = 0.80) measures participants’ eating behavior in relation to negative emotions (e.g., “When I feel lonely, I console myself by eating;” “When I feel anxious, I find myself eating; “When I feel blue, I often overeat”) and has demonstrated reliability and validity [53]. Responses were dichotomized and coded as 0 and 1. The mean emotional eating score from the three items was then calculated, with higher scores indicating a greater level of engagement in emotional eating.

Childhood emotion regulation was assessed by maternal report at age 7 using the Emotion Regulation Checklist (ERC) [54]. The ERC is a 24-item measure that evaluates children’s emotion regulation by caregivers reporting on the frequency of certain child behaviors on a scale 1 to 4 (1 = *never*, 2 = *sometimes*, 3 = *often*, 4 = *almost always*). The *Emotion Regulation subscale* (8 items; α = 0.66) was used to assess children’s emotion regulation skills (e.g., “Is empathetic towards others;” “Can say when she/he is feeling sad, angry or mad, fearful or afraid”). The ERC is a widely used and validated measure of emotion regulation and has been used extensively in previous research with samples of young children [54,55,56].

At age 15, participants completed a lab visit during which their height and weight were measured using standard procedures [49]. Height was measured using a wall-mounted stadiometer and weight was measured using a beam balance scale. For the purposes of the current analyses, participants were classified into three weight status categories in our sample using the BMI-for-age percentiles: (1) healthy weight = 5th to less than the 85th percentile; overweight = 85th to less than 95th percentile; obese = equal or greater than the 95th percentile [57].

Negative body image was assessed using the Body Image and Eating Questionnaire (BIEQ) [58]. The BIEQ is a 14-item measure that was designed to assess specific eating behaviors and body image concerns in youth, with established face validity and good reliability (α ranging from 0.70 to 0.90). For the purposes of the current study, a new 5-item subscale called “negative body image” was created by summing participant responses to the following items: (1) Have you ever thought that you needed to lose weight? Response options: yes/no; (2) Do you think you are fat? Response options: yes/no; (3) I am bothered about how my body looks; response options: never, sometimes, a lot, always; (4) I am bothered because I think I am too fat. Response options: never, sometimes, a lot, always; (5) Do you wish you were thinner? Response options; yes/no. The reliability of the subscale in our sample was high (α = 0.85).

Data from all participants who provided information on the variables of interest during at least two of the three time points were included in the final analyses. Descriptive statistics (i.e., means, SD, frequencies) were used to describe the sample characteristics. Bivariate correlations were utilized to examine the initial associations between the main variables of interest. Multiple regression analyses were performed to examine the interaction effects of childhood emotion regulation, adolescent negative body image and weight status on emotional eating, controlling for SES and gender. To avoid multicollinearity, predictor variables were centered and then multiplied to create interaction terms. Gender and SES were entered as control variables in the first step. In the second step, emotion regulation, negative body image, and weight status were entered, followed by the two-way interactions between emotion regulation and negative body image, emotion regulation and weight status, and negative body image and weight status in the third step. Follow-up tests of significant interactions were probed such that associations between emotion regulation and weight status and negative body image and weight status were examined at the levels of normal weight and overweight/obese status. The associations between emotion regulation and negative body image were examined at low [−1 standard deviation (SD)], mean, and high (+1 SD) levels of negative body image [59]. All statistical analyses were performed using the Statistical Package for Social Sciences (SPSS) (IBM, version 26; Chicago, IL, USA). Statistical significance was set at *p* < 0.05 for all analyses.

## 3. Results

### Sample Characteristics and Main and Moderating Effects

Data from a total of 138 participants were included in the current study. Descriptive statistics and bivariate correlations among study variables can be found in Table 1. The mean SES (15 year) score measured by the Hollingshead index was 45.3 (range 13.5–66.0). The sample was comprised of 52 males and 86 females, with 63% identifying as White, 32% identifying as African American/Black, and 5% identifying as multi-race/other. Ninety-five participants were categorized as normal weight (69%) and 43 participants were overweight/obese (31%) using the established BMI-for-age percentile cut offs [57]. There were no significant gender differences in emotional eating by weight status. However, significant gender differences emerged for negative body image (*t* = −5.95, *p* < 0.001). Females showed significantly higher levels of negative body image in our sample. Interestingly, however, sex-specific correlations revealed only one significant association among study variables: negative body image and emotional eating was significant for males (*r* = 0.44, *p* < 0.05). SES and emotion regulation were correlated (*r* = 0.22, *p* < 0.05). Thus, gender and SES were entered as control variables. Multiple regression analyses were performed to examine the interactive effects of childhood emotion regulation, adolescent negative body image, and adolescent weight status in predicting adolescent emotional eating. The three-way interaction between emotion regulation, negative body image, and weight status was also initially included; however, it was non-significant and so it was removed for parsimony.

**Figure 1:**
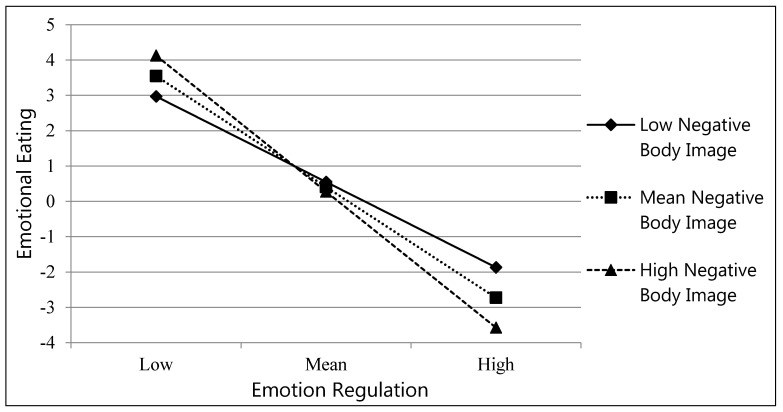
Emotion regulation x negative body image interaction predicts emotional eating.

Main effects were revealed for emotion regulation (*β* = −1.30, *p* < 0.001) and weight status (*β* = −0.28, *p* < 0.05) in predicting emotional eating (Table 2). These effects were qualified by significant interactions between emotion regulation and negative body image (*β* = −0.34, *p* < 0.01) and emotion regulation and weight status (*β* = 1.19, *p* < 0.01). There was also a marginally significant interaction effect between negative body image and weight status (*β* = 0.67, *p* = 0.06) (Table 2). Follow-up analyses revealed that at high (*β* = −1.59, *p* < 0.001) and low (*β* = −1.00, *p* < 0.01) levels of negative body image, the relation between emotion regulation and emotional eating were significant. As emotion regulation increased, adolescents who showed low and high levels of negative body image were less likely to report they were engaging in emotional eating (Figure 1). Additionally, follow-up analyses indicated that for normal weight (*β* = −0.46, *p* < 0.001), but not overweight/obese adolescents (*β* = 0.32, *p* > 0.10), the association between emotion regulation and emotional eating was significant. For normal weight adolescents, as emotion regulation increased, they were less likely to engage in emotional eating. Finally, although the weight status by negative body image was marginally significant, given our hypotheses regarding this association the interaction was probed. Follow-up analyses indicated that for overweight/obese adolescents (*β* = 0.70, *p* < 0.001), but not normal weight adolescents (*β* = 0.19, *p* > 0.10), greater negative body image was associated with more emotional eating.

## 4. Discussion

The primary purpose of the current study was to examine the main and interactive effects of childhood emotion regulation, adolescent weight status and body image in predicting emotional eating at age 17. We found that poor emotion regulation in childhood predicted higher levels of emotional eating engagement in late adolescence and that this association was differentiated by both weight status and negative body image at age 15. While there is a growing body of literature on the role of emotion regulation in obesity development, our study highlights that individuals’ emotion regulation deficits could be targeted in future interventions as early as during middle-childhood as a way to prevent and/or reduce the risk of emotional eating in later adolescence. Our findings also point to the importance of also considering contextual factors during mid-adolescence that may also further shape the influence of emotion regulation on individuals’ reliance on food consumption as a coping mechanism when faced with stress and/or negative feelings during late adolescence. 

Previous research suggests that while emotional eating is fairly low in children, it becomes more prevalent during the adolescent years, and is common among adults and those who are overweight or obese [9,13,60]. Our findings indicate that childhood emotion regulation skills, beginning as early as the elementary school years, have a strong influence on emotional eating in late adolescence. While many experts suggest that emotion regulation may be an effective target for childhood obesity prevention programs [17], most of the current knowledge on emotion regulation and eating behaviors has, so far, come from studies with adults, overweight or obese samples, or clinical populations [60,61]. Thus, findings of the current study provide additional evidence that childhood emotion regulation plays a critical role in shaping later emotional eating, a dysregulated eating behavior that has been closely associated with higher adiposity and increased obesity risk in adolescence and adulthood [5,62]. Furthermore, it builds upon our previous work, which identified that increased engagement in emotional eating is one mechanism by which adolescents who have poor emotion regulation skills are at risk for obesity [5]. 

Despite the growing evidence that emotion regulation plays an important role in the development of healthy eating behaviors and obesity prevention [5,28,60], psychological and other individual-level factors that may influence these associations have not been well studied. The current study extends the existing literature on emotional eating in adolescence by identifying adolescent negative body image and weight status as important factors that influence for whom childhood emotion regulation most strongly predicts emotional eating in late adolescence. In the current study, poor emotion regulation skills were associated with higher levels of emotional eating at both low and high levels of negative body image. However, this effect was stronger among those with a high negative body image, indicating that individuals with better childhood emotion regulation skills are even more protected from engaging in emotional eating when they possess a high body esteem/positive body image by mid-adolescence. Although a number of interventions targeting body image in youth have been developed [63,64,65], further efforts are warranted to develop effective and holistic programs that promote positive body image and prevent body dissatisfaction prior to adolescence [66]. This additional work is especially important in light of previous studies that detected emerging differences in body esteem by gender, weight status and parenting feeding practices in children as young as 6 to 8 years old [67,68].

Existing empirical work with adolescents indicates that body dissatisfaction is prevalent during this unique developmental period, with up to 46% of girls and 26% of boys reporting some degree of dissatisfaction with their bodies [31,69,70]. This is of great concern because body dissatisfaction has been also linked to an increased risk of disordered eating in adolescents [69]. In a study of 2516 adolescents, lower body satisfaction was associated with a variety of unhealthy weight-controlled behaviors, such as the use of diet pills and meal skipping [69]. Binge eating was also correlated with lower body satisfaction in this study, which is not surprising because periods of excessive restriction are often followed by episodes of overeating [69]. In our sample, girls reported a higher negative body image than boys, which was expected and consistent with findings from previous research with this age group [44]. Although the prevalence of negative body image across both sexes tends to increase between childhood and mid-adolescence, interestingly, recent research shows that it remains fairly stable after mid-adolescence. A longitudinal study of 1455 middle- and high-school students who were followed 5, 10 and 15 years later found that body dissatisfaction was relatively stable between mid-adolescence and adulthood for most individuals [44]. In our sample, after controlling for gender and SES, high levels of negative body image at age 15 were associated with greater engagement in emotional eating during late adolescence, but only among overweight/obese individuals. Although the association between body dissatisfaction and BMI and/or weight is complex, our finding indicates that overweight and obese teens are more vulnerable, perhaps via additional peer or parental influences, to the adverse effects of negative body image on their emotional eating [60]. Nonetheless, previous research and the findings of the current study suggest that the critical window for optimizing emotion regulation skills, promoting positive body image and minimizing body dissatisfaction appears to be during childhood and early adolescence [44]. Future obesity and disordered eating prevention efforts should include not only nutrition education, promotion of positive body image and behavioral strategies for healthy lifestyle behaviors, but also emotion regulation education and support so children can learn how to better recognize different emotions, utilize a variety of strategies to manage their emotions and thus avoid using food as a coping mechanism in the future.

The current study contributes significantly to the existing research in this area and has several notable strengths. First, the study utilized data from a diverse community sample of adolescents. The richness of the data is unique and allowed for in-depth examination of the associations between the target constructs of emotion regulation, negative body image, weight status and emotional eating with data collected at different ages. Second, the longitudinal design with multiple data collection time points between ages 7 and 17 allowed us to test main and moderating effects of childhood emotion regulation while also examining the effects of weight status and negative body image in mid-adolescence on emotional eating in late adolescence, which represents a unique approach in the existing literature. The study also had some limitations. The assessment of emotion regulation was based on maternal report and not an observational measure. Emotional eating was based on participants’ self-report and thus was subject to a certain level of bias. However, the adolescent subscale of emotional eating utilized in the study, from the Three Factor Eating Questionnaire (TFEQ), was validated and used in previous studies with similar sample characteristics. Negative body image in the current study was assessed using a new subscale created from the Body Image and Eating Questionnaire (BIEQ) items that were administered as part of the original longitudinal study. Further research on negative body image in this population should examine this construct utilizing one of the existing multi-dimensional measures of body image. Given the differences in societal standards of beauty for females and males, the use of gender-specific measures of body image and/or body satisfaction is warranted in future research to better understand how gender influences emotional eating and other food- and weight-related behaviors. Given the limitations of our sample, additional analyses related to race/ethnicity were not feasible in the current study. However, given the reported differences in obesity risk as well as body satisfaction between racial/ethnic groups in the U.S., future investigations are warranted to examine the role of race/ethnicity in the development of adolescent emotional eating [31,71,72]. Lastly, parental and peer variables and additional personal constructs (i.e., history of dieting) were not considered in the models that were tested and future research should examine these as they may play important roles in the development of emotional eating in adolescence.

## 5. Conclusions

Obesity continues to represent a major public health issue in the U.S. [71,72]. Recent projections suggest that nearly 50% of today’s children will become obese by age 35 [73]. At the same time, emotional eating has been established as one of the contributing factors to unhealthy dietary patterns and increased obesity risk among children and adolescents [5,29,47]. Because lifestyle habits, including eating behaviors, continue to be shaped into young adulthood, it is critical that we develop a better understanding of factors that may prevent emotional eating in youth. The current study shows that development of high emotion regulation skills by middle-childhood, early prevention of overweight/obesity and promotion of body satisfaction represent positive cognitive, behavioral and/or psychological factors that may protect children from engaging in emotional eating later in adolescence. To date, most obesity prevention and treatment programs in youth have had limited success, largely focusing on calorie-reduced diets and/or increasing physical activity [9]. While these are important components of healthy weight management programs, ongoing interdisciplinary research has made it increasingly clear that future interventions must also include specific components that address key cognitive, behavioral and psychological processes related to eating behaviors and psychological health [9,29,74]. The findings of the current study provide additional evidence for such steps and highlight the importance of childhood emotion regulation, weight status and negative body image in the development of dysregulated eating in late adolescence.

## Figures and Tables

**Table 1:** Bivariate correlations and descriptive statistics.

	1	2	3	4
1. 15 year SES ^a^	--			
2. 7 year Emotion Regulation	0.22 *	--		
3. 15 year Negative Body Image	−0.10	0.01	--	
4. 17 year Emotional Eating	0.08	−0.02	0.25 **	--
Mean	45.26	3.42	0.00	0.41
Standard Deviation	12.91	0.35	0.83	0.85
Minimum	13.50	2.75	−0.87	0.00
Maximum	66.00	4.00	1.88	3.00

Note: ** *p* < 0.01, * *p* < 0.05; ^a^ socioeconomic status.

**Table 2:** Regression analysis on associations between 7 year emotion regulation, 15 year weight status, 15 year negative body image, and 17 year emotional eating.

	B	SE (B)	β
17 year Emotional Eating ^a^			
Sex	0.11	0.18	0.07
15 year SES ^b^	0.01	0.01	0.13
7 year Emotion Regulation	−3.14	0.86	−1.30 ***
15 year Negative Body Image	−0.19	0.33	−0.19
17 year Weight Status	−0.52	0.24	−0.28 *
7 year Emotion Regulation × 15 year Negative Body Image	−0.98	0.33	−0.34 **
7 year Emotion Regulation × 17 year Weight Status	2.19	0.66	1.19 **
17 year Weight Status × 15 year Negative Body Image	0.47	0.25	0.67 ^+^

Note: ^a^ Outcome Variable; *** *p* < 0.001, ** *p* < 0.01, * *p* < 0.05, ^+^ *p* < 0.10; ^b^ socioeconomic status.

## Data Availability

The data presented in the current study are not publicly available due to the stipulations related to privacy and confidentiality in the approved IRB protocol. However, the data are available upon request from the corresponding author (LHS).
